# Adrenomedullary Organoids Derived from Neural Crest and Schwann Cell Precursors

**DOI:** 10.21203/rs.3.rs-10607254/v1

**Published:** 2026-08-11

**Authors:** Hsueh-Fu Wu, Jessica McAlpine, Christina James, Tripti Saini, Piyush Padhi, Samantha D. Baxley, Casey L. Stewart, Dong Eun Seo, Mo-Fan Huang, Heidi Ulrichs, Jennifer Art, Fabio R. Santori, Anumantha G. Kanthasamy, Dung-Fang Lee, Natalia B. Ivanova, Oshri Avraham, Nadja Zeltner

**Affiliations:** University of Georgia; University of Georgia; University of Georgia; University of Georgia; University of Georgia; University of Georgia; University of Georgia; University of Georgia; The University of Texas Health Science Center at Houston; University of Georgia; University of Georgia; University of Georgia; University of Georgia; The University of Texas Health Science Center at Houston; University of Georgia; University of Georgia; University of Georgia

**Keywords:** sympathoadrenal progenitor, Schwann cell precursor, chromaffin cells, adrenal glands, organoids, human pluripotent stem cells

## Abstract

The adrenal gland, essential for survival, contains the medulla and cortex. Chromaffin cells (ChrCs) in the adrenal medulla control the fight-or-flight response by releasing catecholamines like epinephrine and norepinephrine. Recent findings reveal that ChrCs originate from two progenitors: 20% from developmentally early, neural crest-derived sympathoadrenal progenitors (SAPs) and 80% from the later Schwann cell precursors (SCPs). We generated functional adrenomedullary organoids from human pluripotent stem cells (hPSCs) via both pathways and in mixed (20% SAP, 80% SCP-derived) cultures. When implanted into chicken embryos, the organoids migrated and integrated into the adrenal gland anlagen. *In vitro*, they released norepinephrine and epinephrine in response to stimulation. Single-cell transcriptomics confirmed developmental trajectories matching human fetal data and allowed the identification of a novel ChrC subtype specifically derived from the SAP that may be responsible for mediating the severe stress reaction via EPI. Assessing developmental knowledge in this human system, we found that SAPs differentiated into ChrCs, but not sympathetic neurons or Schwann cells, showing limited plasticity. Meanwhile, SCPs could produce various cell types but not sympathetic neurons. Furthermore, our work reveals that under stress conditions, SAP-derived ChrCs mainly release epinephrine, while SCP-derived ChrCs release norepinephrine, highlighting distinct functions based on developmental origin. These organoids provide valuable models for studying ChrC development, interactions, and adrenal pathologies.

## Introduction

The adrenal gland is a vital endocrine organ that regulates the body’s homeostasis and the stress response. The postnatal adrenal gland has a layered anatomy that is composed of the medulla, cortex, and capsule. The medulla, containing chromaffin cells (ChrC), is located in the core of the adrenal gland. It is surrounded by a multi-layered adrenocortical shell, containing three different endocrine cell types, composed inside out of the zona Reticularis (zR), the zona Fasciculata (zF), and the zona Glomerulosa (zG). The organ is finally wrapped in a capsule^1^. The adrenal gland also contains adult stem cells for both the adrenal cortex and adrenal medulla. Namely, the capsule and zG harbor capsular and subcapsular stem cells, respectively, which govern adrenal cortex self-renewal throughout life^2^. Glia-like stem cells, called sustentacular cells are spread throughout the entire gland with some evidence suggesting that they differentiate into ChrCs upon stress^3^. ChrCs release catecholamines: epinephrine (EPI, also called adrenaline) and norepinephrine (NE, also called noradrenaline), both mediating the acute stress response^4^. The hormonal output of the adrenal medulla is approximately 80% EPI and 20% NE^5^. Adrenal gland cortical zR cells release androgen precursors (for production of sex hormones in the ovary and testis during both development and adulthood), zF cells release glucocorticoids (for the stress reaction; stimulates gluconeogenesis, inhibits mediators of inflammation) and zG cells release mineralocorticoids (mediating blood pressure regulation and plasma Na^+^ and K^+^ concentrations)^6^.

During human embryonic development, the adrenal gland develops from two independent germ layers. Namely, the cortex is derived from the mesoderm, whereas the medulla is derived from the ectoderm, specifically the neural crest. Our knowledge about the adrenomedullary development has shifted in recent years. Before 2017, a large body of work, mostly in the mouse, had postulated that the neural crest gives rise to sympathoadrenal progenitor (SAP) cells, which then develop into both sympathetic neurons of the sympathetic chain and adrenomedullary cells^7,8^. However, this view has been revised both in the mouse and in humans^9–11^. It is now thought that the medulla is derived from two distinct, but both neural crest-derived progenitors. Namely, about 20–25% of the medulla is derived from the SAP early on in development. The remaining 75–80% of the medulla is derived later in development from the Schwann cell precursor (SCP, Fig. 1a). The SOX10^+^ neural crest is a transient, embryonic, highly migratory, multipotent stem cell population, giving rise to various cell types^12^. At around 7 wpc, SAP invade the adrenal gland and settle in its center in diffuse clusters^13^. These progenitors are marked by *ASCL1, PHOX2B*, and *GATA2/3* and give rise to about 20–25% of the final medulla. The other 75–80% of ChrCs are derived from the SCP, which is a multipotent, embryonic cell type derived from the early neural crest, with the capacity to give rise to many cell types, including melanocytes, parasympathetic neurons, tooth pulp cells and odontoblasts, enteric neurons, chondrocytes, and osteoblasts^14^. Pre-ganglionic nerves innervate the adrenal gland, and SCPs migrate along these pre-ganglionic nerve tracks and populate the adrenal gland medulla, where they differentiate into ChrCs^15^. ChrCs can be adrenergic (adrenaline/EPI releasing) or noradrenergic (noradrenaline/NE releasing)^16–18^. However, the developmental origin of these functional subgroups has not been elucidated. Particularly, it is not clear whether each progenitor (SCP and SAP) preferentially gives rise to either the noradrenergic or adrenergic phenotype.

There are significant differences between the human and mouse adrenal gland. Several studies assessing human fetal adrenal gland transcriptomes in bulk or on the single cell level have improved our understanding of human adrenal development in comparison to the mouse. Furthermore, single cell transcriptomics (scRNA-seq) studies have revealed differences between human and murine ChrC development regarding the developmental potential of intermediate cell states. RNA velocity analyses have shown that murine intra-adrenal sympathoblasts differentiate from ChrCs^19^. However, human ChrCs and intra-adrenal sympathoblast differentiation potential was shown to be bi-directional, with sympathoblasts differentiating from ChrCs and vice versa. Furthermore, in humans, SCPs were shown to differentiate into both ChrCs and immature sympathoblasts, whereas in mice SCPs were shown to differentiate only into ChrCs^19^. Importantly, these publications have also shed light on markers of human adrenal progenitor cells throughout their stages of development, which differ from those in mouse as well^19–21^.

The adrenal gland in humans can be affected by a myriad of diseases^22^. Interestingly, most common adrenal gland disorders mainly affect the cortex, including adrenal insufficiency, Cushing’s syndrome, and tumors. Diseases of the medulla are rare, possibly indicating its absolute necessity for life.

Examples of diseases derived from medullary cells include neuroblastoma, the most common solid, non-brain cancer in young children, which was shown to arise from SAPs^23^. Pheochromocytoma, is a rare tumor of the ChrCs residing within or outside the adrenal gland medulla^24^. As these tumors release excessive amounts of catecholamines, patients present with stress-related, psychiatric symptoms^24^. Accordingly, ChrCs are essential for the regulation of the stress reaction. ChrCs are also analgesic, for example ChrC implants have been tested to alleviate cancer pain^25^, as they release analgesic peptides^26^. This highlights the prevalence of human disorders caused by adrenal gland dysfunction and calls for regenerative strategies of the gland. Thus, having isolated, human, untransformed ChrCs in hand would enhance research into various human disorders, may facilitate drug screening/testing approaches for the development of novel treatments and will enhance our ability to dive deeper into the molecular mechanisms driving development of the adrenal gland.

The human pluripotent stem cell (hPSC) technology is a powerful tool that allows the generation of specific human cell types in the dish. Many *in vitro* differentiation protocols showed the derivation of human neural crest from hPSCs^27–29^. To our knowledge only three papers have demonstrated the derivation of adrenal gland ChrCs from hPSCs^30–32^. Abu-Bonsrah et al. differentiated hPSCs into SAP-derived ChrC-like cells, using WNT activation and TGFβ inhibition early, followed by FGF2 and BMP2 mediated expansion, followed by final differentiation mediated by treatment with BMP4, dexamethasone (a glucocorticoid), and phorbol 12-myristate 13-acetate (PMA, a PKC activator)^30^. The ChrC-like cells produced by this differentiation paradigm express appropriate markers, released relatively low levels of both NE and EPI, and showed some migration and *in vivo* integration into the chick. However, recent discoveries in adrenomedullary development, i.e. the large contribution of SCP to the ChrC population, as well as gene expression patterns discovered in human fetal adrenal gland tissue could not be addressed at the time. Thus, it is likely that the ChrC-like cells derived by Abu-Bonsrah et al. may be similar to the ~ 20–25% of ChrCs derived from the early neural crest/SAP, but not the remaining cells derived from the SCP. Van Haver et al. also differentiated hPSCs into sympathoblasts (the intermediate between the sympathoadrenal progenitor and chromaffin cells) to study neuroblastomas, which are derived from this intermediate^31^. However, they claimed that a modification of their differentiation to include a longer duration of BMP4 exposure would result in chromaffin cells rather than sympathoblasts, but they did not elaborate on this, as chromaffin cells were not the focus of their story. Saldana-Guerrero et al. detailed a directed differentiation of trunk neural crest cells to various derivatives^32^. One of which has a partial chromaffin identity, with chromogranin A expression but no PNMT (an enzyme important for epinephrine synthesis). Because this differentiation results in a mixed population of trunk neural crest derivatives, it is not easily used to study the chromaffin cells themselves. Furthermore, as there are sympathoadrenal progenitors and Schwann cell precursors in this mixed population, it is difficult to distinguish which progenitor these partial chromaffin cells came from, hindering any developmental analysis of these chromaffin cells. In sum, there is a lack of a directed differentiation protocol that generates a pure population of functional chromaffin cells from either progenitor of the adrenal medulla.

Here, our goal was to generate adrenomedullary organoids from hPSCs and employ the technology to assess recent developmental findings about the two sources of ChrCs and the developmental potential of their progenitors in the human system. We showed that neural crest can be differentiated into SAP and can generate differentiated ChrCs. Similarly, hPSC-derived neural crest cells can generate SCPs^33^, which then also can differentiate into ChrCs. ChrCs from both sources were functional and released NE and EPI upon stimulation. We conducted scRNA-seq analysis on both SCP-derived ChrCs and SAP-derived ChrCs and found that they gave rise to different subtypes of ChrCs. When injected into the chick embryo, the ChrC progenitors migrated appropriately to the mesonephros or integrated into the chicken adrenal gland. We then employed this system to assess human developmental paradigms by testing the differentiation potential of the respective progenitors. These cell types will be instrumental for modeling and studying various diseases that affect the adrenal gland, improving the possibility to elucidate novel treatment methods in the future.

## Results

### Generation of adrenomedullary organoids from sympathoadrenal progenitors.

We first set out to generate the SAP progenitors from hPSCs. During embryogenesis, early migrating neural crest cells express the markers *SOX10*, *CD49D* and *FOXD3*^34^. Once they aggregate at the dorsal aorta, in response to BMP signaling^35^, expression of *PHOX2B*, *ASCL1*, *INSM1*, *HAND2* and *GATA2/3* is induced^36^, priming them to further differentiate into ChrCs (the 20–25% of the medulla, Fig. 1A, purple cells). At that stage their axial HOX code, marking the A-P axis, also shifts to a trunk identity, marked by expression of HOX genes from *HOX2* to *HOX7*^37^. We have used a previously established protocol^38,39^ to differentiate neural crest cells with a cranial character. It has previously been shown that neural crest cells can be posteriorized with retinoic acid (RA)^28^ to form vagal neural crest, which increases their HOX code. Once neural crest cells aggregate near the dorsal aorta, they receive BMP4^35^, SDF1 and NRG1^7^ signals. Thus, we reasoned that RA will provide a trunk-like character^28^, and that BMP^35^, NRG1 and SDF1^7^ were important factors that prime these neural crest cells to become ChrCs. Indeed, compared to cranial neural crest^38^ and vagal neural crest^28^, our SAP expressed a more posterior HOX code (HOXB2/4/5/7), as well as higher levels of the SAP markers, including *ASCL1, PHOX2B* and *HAND2*, but not the unrelated sensory neuron marker *BRN3A* (data not shown). We then tested if starting the ChrC-priming factors (RA, SDF1, NRG1, BMP4) at day 4, 5, or 6 results in higher expression of these markers. We found day 4 to be ideal in terms of levels of *HOXB7* and *HAND2* expression (data not shown). Thus, going forward, we settled on this protocol (Fig. 1B). Morphological analysis of the cells using this protocol shows typical cell morphology, such as arrangement into dense neural crest ridges (arrows, day 10, Fig. 1C). These SAP cells expressed *SOX10, CD49d*, and *FOXD3* (Fig. 1D), starting at day 4. SOX10 expression went down with proceeding time, consistent with the early neural crest differentiating into SAP. By day 10, *HOXB2/5/7*^37^ were expressed the strongest (Fig. 1E). By day 10, *ASCL1, PHOX2B, and GATA2* were strongly expressed consistent with an SAP identity (Fig. 1F, G).

Next, we aimed at further differentiating the SAP into ChrCs. After a four-day spheroid expansion period (day 10–14, Fig. 2A, B)^39^, we assessed both adherent 2D or continued 3D cultures for ideal differentiation into ChrCs in a WT hPSC and a control induced pluripotent stem cell (iPSC) cell line (**Figure S1A**, hPSCs-ctr-H9 (top) and iPSC-ctr-C1 (bottom)). Based on the available literature, we designed a ChrC media cocktail comprised of BMP4^30^, ascorbic acid (AA)^40^, NRG1^15^, IGF2^41,42^, NGF^42–44^, FGF2^42,45^, dexamethasone^44,46^ and PMA^47^ for the differentiation and maturation of ChrCs. We found that adherent 2D cultures did not express the ChrC markers *PNMT* and TH at day 30, while floating 3D cultures did (Fig. 2C, S1B, C). Using the 3D condition, we found that the cells downregulated expression of *ASCL1* and *PHOX2B* (Fig. 1F) and upregulated expression of *GATA2/3* and *INSM1* (Fig. 1G) from day 10 to 20 consistent with early ChrC development^48^. *In vitro* cultures can be defined as organoids when they meet several of the following criteria: 1) they are derived from a stem or progenitor cell type, 2) the cultures are maintained in 3D, 3) the cultures contain several cell types of the native organ, 4) there are some structural and functional characteristics present that mimic the native organ. Based on our 3D data and data that will follow in this work, from here on we call our ChrC cultures, adrenomedullary organoids. For an easy and convenient differentiation protocol, it is useful to have a cryopreservation step that allows the generation of the cells in a shorter period from previously frozen and well characterized cell stocks. We found that SAPs can be cryopreserved and successfully thawed at day 10 with above 60% post-thaw viability (**Figure S1D**).

We then went on to a deeper characterization of these adrenomedullary organoids (Fig. 2C). Between day 20 and 30, the cells mature further and start expressing early ChrC markers, including *DLK1* and *FOXQ1* (markers found in the mouse early ChrC lineage^49^ (Fig. 2D). High magnification of day 30 ChrCs shows classic chromogranin granules, as seen in primary chromaffin cultures^50^ (Fig. 2E). We found that the cells also expressed mature markers, including the enzymes necessary for catecholamine synthesis, such as *TH, DBH, PNMT* and the glycoprotein chromogranin B (*CgB*, Figs. 2F). Additionally, immunofluorescence confirmed the presence of these enzymes and revealed that the adrenomedullary organoids produce chromogranin A (CgA) as well as both EPI and NE (Fig. 2G).

ChrCs work closely with the endocrine and sympathetic nervous system to respond to acute stress. In stress, the splanchnic nerve stimulates ChrCs with acetylcholine (ACh) and PACAP, inducing a calcium influx that results in EPI and NE release. Catecholamine release is also regulated via autocrine and paracrine regulation such as histamine^51^, glucocorticoids, angiotensin, opioids or GABA^52,53^. To address our adrenomedullary organoids’ functionality, we first used Ca^2+^-imaging to assess their reactivity upon stimulation with ACh, which sympathetic neurons innervating the adrenal gland would release to stimulate catecholamine release^54^, or KCl and found increased Ca^2+^ influx upon stimulation (Fig. 2H).

To further assess the adrenomedullary organoids’ ability to release classical neurotransmitters, we used a standard panel of metabolites in HPLC. We found that they released L-DOPA, NE, EPI, DOPAC, DA, 5-HIAA, HVA, 3-MT, and 5-HT (Fig. 2I). The catecholamines crucial to chromaffin cell function (NE and EPI) are prone to oxidation and rapid metabolic breakdown, leading to an increase in additional peak areas in the HPLC and to an increase in deaminated metabolites (DOPAC, HVA, and HIAA). Therefore, the height of these metabolite peaks in the HPLC can potentially be attributed to NE/EPI deamination.

It is known that factors released from ChrCs stimulate adrenocortical cells to release cortisol^55^ and contribute to maturation and organization of adrenal cortical cells^48,56^. To assess if our adrenomedullary organoids have this functional capacity, we cultured the adrenocortical cancer cell line H295R^57^ with conditioned medium from adrenomedullary organoids and measured their release of cortisol after 48 hours via ELISA (Fig. 2K). Treating H295R cells with ChrC conditioned media induced them to release >5ng/mL of cortisol (Fig. 2K). As a positive control, we stimulated H295R cells with not conditioned ChrC media containing forskolin (FSK)^57^, eliciting a very strong release of ~ 14ng/mL cortisol. Finally, we measured changes in steroidogenic potential of the H925R cells upon treatment with the ChrC-conditioned media and found increased expression of the steroidogenesis markers *STAR* and *CYP11B1*, which are needed for cortisol synthesis (Fig. 2L). This suggests that hPSC-derived adrenomedullary organoids are capable of paracrine stimulated of cortisol secretion in adrenal cortex cells.

### Generation of adrenomedullary organoids from Schwann cell progenitors.

We next aimed at employing this system to assess developmental findings within the ChrC lineage, that has been reshaped in recent years. It is now known that early neural crest cells give rise to nerve-associated SCPs, which subsequently travel along established preganglionic nerve tracks and populate up to 80% of the adrenal medulla as SCP-derived ChrCs^15^ (Fig. 1A, teal cells). Thus, we aimed at assessing if hPSC-derived SCPs had the capacity to generate adrenomedullary organoids *in vitro*. We employed a SCP differentiation paradigm that has been established recently by Majd et al., where it was shown that these cells can further be differentiated into Schwann cells that successfully myelinate neurons^33^. There, neural crest cells are first generated as previously described^38,39^, followed by a 6 day spheroid culture period, supplemented with FGF2 and WNT (which maintains the neural crest cell state^28^), as well as NRG1, the most critical SCP inducing and survival factor^36^. Figure 3A shows that, accordingly, in our hands, SCPs are produced in 16 days, based on expression of the SCP markers *EGR2, DHH, CDH19, GAP43, PMP22* and *PLP1*. At day 16, the cells express SCP protein MPZ by immunofluorescence (Fig. 3B), and the SCPs are capable of further differentiating into Schwann cells expressing MBP and they myelinate nanofibers one week after they were plated in the nanofiber dishes (Fig. 3C). We have previously used these SCPs to generate parasympathetic neurons in Wu et al^58^. With these bonified SCP cells in hand, we then cultured day 16 SCPs in the ChrC differentiation and maturation media established earlier and assessed their developmental capacity to generate ChrCs. The differentiation scheme from SCP to ChrCs is detailed in Figs. 3D, and differentiation snapshots from days 0 to 30 are shown in Fig. 3E. SCP-derived adrenomedullary organoids expressed the appropriate ChrC markers CgA, TH, EPI, PNMT and NE (Fig. 3F, via immunofluorescence). They also expressed appropriate intermediate markers (*FOXQ1* and *DLK1)* (Fig. 3G) and mature markers (*TH, DBH, PNMT*, and *CgB*) at the mRNA expression level (Fig. 3H). Similar to SAP-derived ChrCs, as expected, the SCP-derived ChrC-conditioned media was capable of stimulating H295R cells to release cortisol (Figs. 3I and 3J) and increased expression of steroidogenesis markers *STAR* and *CYP11B1* (Fig. 3K). Together, this data confirms that SCPs have the potential to generate ChrCs *in vitro* in the hPSC system, similar to findings in the mouse^15^.

Since the adrenal gland medulla is made up of ChrCs derived from progenitors of the SAP and the SCP, we wanted to assess if *in vitro* mixing of these two progenitors may lead to a functionally superior medulla organoid. Thus, we mixed the two progenitors (the SCP constitutively expressing RFP) at a ratio of 80% SCP:20% SAP (**Figures S2A and S2B**), let them develop into adrenomedullary organoids and assessed their functionality. **Figures S2C and S2D** shows that the mixed adrenomedullary organoids express the appropriate markers (*CgB* and *TH*) comparably to each of the individual progenitor cellderived adrenomedullary organoids. Similarly, conditioned media from the mixed adrenomedullary organoids was able to stimulate cortisol release (**Figures S2E and S2F**), and increased gene expression of steroidogenic genes in H925R adrenocortical cells (**Figure S2G**). This data shows that mixed progenitor culture organoids give rise to ChrCs appropriately, but we were not able to detect character or functional differences in mixed progenitor adrenomedullary organoids compared to individual progenitor adrenomedullary organoids. It is possible that further maturation time would allow such differences to be detected.

### Single-cell transcriptomics analysis of adrenomedullary organoids

To further delineate the transcriptomic identity and developmental trajectory of the adrenomedullary organoids derived from either the SAP or SCPs, we subjected day 30 organoids to scRNA-seq analysis. The organoids were differentiated from SAP or SCPs, individually sequenced, and then subjected to quality control, processing, and integration using standard Seurat workflow (Figs. 4A, S3A). Cluster annotation was performed using a panel of genes for medulla cell type markers from a variety of developmental stages. Figure 4B and 4C show the cell type composition of our adrenomedullary organoids, containing a total of 14 clusters. Four clusters of ChrCs (named ChrC1-ChrC4: ChrC1, *AGTR1*^+^/*PNMT*^+^/*CLDN11*^+^; ChrC2–3, *CHGA*^+^*/DBH*^+^*/TH*^+^; ChrC4, *PNMT*^+^/*ADM*^+^), two sympathoblast clusters (named proliferating and non-proliferating, *STMN2*^+^/*ISL1*^+^/*PRPH*^+^), and one cluster of cells with overlapping ChrC/sympathoblast expression (named intermed. ChrC/Symp., *STMN2*^+^/*ELAVL2*^+^*/ADM*^+^*/CHGA*^+^). Additional identified cell type clusters included early ChrC/bridge cells (named ChrC/bridge, *ASCL1*^+^/*PHOX2B*^+^/*INSM1*^+^/*DBH*^+^), three clusters of transcriptionally distinct progenitor cells (named progenitor 1 (*SOX5*^+^/*SOX*6^+^ /*ANK3*+/*CLDN11*^+^), progenitor 2 (*SOX5*^+^/*SOX*6^+^/*NELL2*^+^), and proliferating progenitor (*RTTN*^+^/*SOX5*^+^/*SOX10*^−^), SCP/Progenitor cells (*SOX10*^low^*/PLP1*^low^/*ASCL1*^*low*^), cycling SCPs (*PLP1*^+^/*SOX10*^+^/*MPZ*^+^), and an unknown cluster. While some of the four ChrC clusters share certain features, they vary in differential expression of multiple signaling peptides and processing enzymes (Figs. 4C (box), **S3B**). While ChrC2 and ChrC3 expressed canonical ChrC markers such as *TH*, *DBH*, and *CHGA/B*, expression of *CARTPT* was found only in ChrC3. Notably, *PNMT* expression was absent both in ChrC2 and ChrC3. *PNMT* expression, necessary for EPI production, was found both in ChrC1 and ChrC4, while *TAC1* expression was exclusive to ChrC4, and *AGTR1* was expressed solely in ChrC1. While ChrC1 and ChrC4 did not express most canonical ChrC markers (such as *TH, DBH, CHGA/B*), they were identified as ChrCs based on PNMT expression combined with the absence of sympathoblast and SCP gene expression. To understand the origin of each identified cell type, we next colored the UMAPs by their sample origin (Figs. 4D, S3C) and quantified the percent sample representation of each cluster (Fig. 4E). We found that clusters ChrC2 and 3 are almost entirely derived from SCP cells (97.4% and 99.2%, respectively), as well as the sympathoblasts (95.5%), bridge/early ChrC (96.5%), proliferating sympathoblasts (94.8%), cycling SCP (70%), and ChrC4 (71.0%, Fig. 4D, 4E, blue). In contrast, ChrC1 was the only cluster with a large majority of SAP origin (84.5%, Fig. 4D, 4E, pink). Progenitors 1 and 2 were made up of mixtures of the two progenitors (60.9% SCP, 67.8% SCP, respectively, Fig. 4E). This suggests that SAP cells give rise to ChrC1 cells. It further suggests that SCP cells are the origin of the ChrC2 and ChrC3 cells, which do not express *PNMT*, thus do not produce EPI. Overall, these trajectories recapitulate findings in recent publications that both SAP and SCP give rise to ChrCs^10,19,31^. We next performed trajectory analysis using Monocle3 (Fig. 4F), setting the root in the UMAP region with the highest expressions of both *PLP1* and *ASCL1* (Fig. 4G) that is comprised of a mixture of SAP and SCP. Trajectory analysis revealed that the progenitors began by sharing similar transcriptional profiles but gave rise to diverging trajectories. This results in ChrC clusters that have disparate transcriptomes depending on their progenitor of origin as indicated by the dashed lines in Fig. 4F.

Next, we assessed how our hPSC-derived adrenomedullary organoids relate to the human embryonic adrenal gland. We re-analyzed the transcriptomics data by Wang et al, derived from human gestation week (GW) 9 embryonic adrenal gland tissues^21^ (**Figure S3D**). Clusters were called based on differential gene expression (**Figure S3E**). We then identified the medulla clusters (i.e. sympathoblasts and proliferating sympathoblasts, *ISL1*^+^/*PRPH*^+^; SCPs, *PLP1*^+^/*SOX10*^+^; intermediate ChrCs/sympathoblasts; ChrCs *CARTPT*^+^) and re-clustered them for a renewed focus on the fetal medulla only (Fig. 4H, I, S3F). Upon re-clustering, larger clusters were broken up into smaller clusters representing transcriptionally distinct cellular subtypes (Fig. 4H, I). These new, smaller clusters include bridge/early ChrCs (*ASCL1*^+^/*ADM*^+^/*NELL2*^+^), as well as two clusters originally defined as intermediate ChrC/Sympathoblasts (**Figure S3D, E**), one differentially expressing *CLDN11* and *AGTR1*, and another differentially expressing *DLK1* and *ERGIC3* (known ChrC progenitor markers^19^ (Fig. 4I). We then performed correlation analysis on the transcriptional profiles of our SAP- and SCP-derived adrenomedullary organoids compared to the re-clustered GW9 embryonic data (Fig. 4J, S3G). This correlation analysis revealed that the embryonic ChrC cluster most strongly correlated with the organoid cluster ChrC3, followed by cluster ChC2. We then highlighted which developmental origin these highly correlating clusters have and showed that embryonic ChrC/organoid cluster ChrC3 is derived from SCP (99.2%, Fig. 4K). We further found that the *CDLN11*^+^/*AGTR1*^+^ embryonic ChrC-like cluster correlated most strongly with the ChrC1 organoid cluster (Fig. 4J), whose transcriptional profile is distinct from the canonical ChrC marker-expressing clusters ChrC2 and ChrC3. Furthermore, ChrC1 is the only organoid ChrC cluster originating primarily (84.5%) from SAP-derived organoids (Fig. 4D, E, J, K).

To further investigate the relationship between clusters ChrC1 (organoid) and *CLDN11*^+^/*AGTR1*^+^ (embryonic), we compared their differential expression profiles and found that of those conserved genes, the majority were mutually upregulated, indicating a highly similar differential gene expression profile (**Figure S4A**). Furthermore, the top 10 differentially expressed genes from each group are largely exclusively differentially expressed in the *CLDN11*^+^/*AGTR1*^+^ cluster compared to the other embryonic medulla clusters (**Figure S4B**, box). A *CLDN11*^+^ chromaffin-like cell type has been identified previously by Bedoya-Reina et al., who described a *CLDN11*^+^/*NTRK2*^+^ post-natal chromaffin progenitor (*SOX6*^+^/*RTTN*^+^) with an adrenergic transcriptional signature (*CHRNA7*^+^). These *CLDN11*^+^/*NTRK2*^+^ cells did not express canonical ChrC markers (such as *TH, DBH, CHGA/B*) and had distinct morphology and distribution within the adrenal gland compared to the rest of the adrenal medulla. In our adrenomedullary organoid dataset, *NTRK2* is almost exclusively observed in the Progenitor 1 cluster (**Figure S4C**, top), which is located directly adjacent to ChrC1 on the UMAP (Fig. 4B). *CHRNA7* is expressed most highly in ChrC2 and ChrC3, but still expressed at low levels in both Progenitor 1 and ChrC1, similar to Bedoya et al.’s observations. Furthermore, Progenitor 1 expresses many progenitor markers described by Bedoya-Reina et al. in addition to *NTRK2* (*RTTN*, *BCL11A*, *SOX6*; **Figure S4D)** and is comprised of a large number of SAP cells, which may be the progenitors of the *CLDN11*^+^/*AGTR1*^+^ population. Together, these results recapitulate the findings of studies identifying SCPs as the origin of the majority of ChrCs and sympathoblasts and indicate the identification of a novel ChrC-like cell type in the human adrenal medulla, which may arise from the SAP progenitor (Fig. 4L).

### Human pluripotent stem cell-derived chromaffin cell progenitors integrate into the chicken embryo.

We aimed at corroborating some of our *in vitro* findings *in vivo*. We generated SAP from a hPSC line that constitutively expresses RFP for 14 days and transplanted the differentiated or non-differentiated (day 0) cells as a control into HH16 stage chick embryos (Fig. 5A). Cells were injected into the intersomitic space of the trunk region (Fig. 5B). Trunk neural crest cells have distinct migratory routes from the dorsal neural tube; the ventromedial pathway mainly drives neural crest cells towards sympathoadrenal fates (neurons and glia of the sympathetic ganglia and ChrCs of the adrenal medulla), the ventrolateral path will contribute to sympathetic fates, and to the dorsal root ganglia neurons and the Schwann cells of the ventral roots. The dorsolateral pathway drives neural crest cells dedicated to the generation of pigment cells of the epidermis^59^. 36 hours after injecting the cells, at HH22, the embryos were sacrificed and sectioned to assess if the injected cells migrated to the appropriate regions within the embryo. We found that, while the undifferentiated cells (day 0) were found in several locations in the embryo, including the neural tube (Fig. 5C, 1, arrowheads), the mesonephros (2), the wing/limb (3) and skin (4), the SAP (day 14) integrated mainly to the mesonephros/adrenomedullary area (Fig. 5C, 2). As an additional control, we injected RFP^+^ cranial neural crest cells that were differentiated for 10 days and are expected to give rise to structures in the head and face. Cranial neural crest RFP^+^ cells were not detected in the mesonephros, or generally in the trunk area, and were found predominantly in the head around the myencephalon (5) and the optic vesicles (6, Fig. 5D).

To assess if hPSC-derived, more mature ChrCs integrate into the chicken tissue, we generated SAP-derived adrenomedullary organoids and injected them at day 30 into HH23 chick embryos, targeting the back on top of the mesonephros, where the adrenal gland is located (Figs. 5E and 5F). 36 hours later the embryos (HH27) were sacrificed and sectioned for immunofluorescence imaging. We co-stained the sections with a human specific antibody, HNA and EPI. We found that the chick mesonephros/adrenal medulla area, contained human cells that were also positive for EPI (Fig. 5G), suggesting that they integrated into the tissue, as prerequisite for potential functionality in the chick embryo. In sum, this data confirmed that the ChrC progenitors and ChrCs have the capacity to integrate into the appropriate location *in vivo*.

### Investigation of the developmental potential of the sympathoadrenal progenitor cells.

The hPSC differentiation system may be considered somewhat artificial and may not always perfectly represent embryonic development as it occurs *in vivo*. Nevertheless, the following reasons lead us to assess developmental paradigms in the human system using this hPSC technology: (i) Human embryonic developmental events are difficult or impossible to study *in vivo* for ethical and lack of access to tissue reasons. (ii) Developmental pathways from neural crest, SAP and SCPs to various structures are currently under intense investigation in various *in vivo* models^60–62^ (**Figure S5**). (iii) Different progenitors may have dynamic developmental potential, allowing them to generate several progeny cell types. We first asked if our SAP cells *in vitro* can generate sympathetic neurons, which would place them characteristically closer to the often-described bipotent SAP^7,8^ (Fig. 6A). We generated day 10 SAP cells and cultured them in sympathetic neuron differentiation media that was previously established^39^ (Fig. 6B). Wu et al. generated sympathetic neurons at day 30, at 45% efficiency (i.e. 45% of all cells are PRPH^+^ peripheral neurons) and over 90% specificity^63^ (i.e. 90% of the PRPH^+^ peripheral neurons are also TH^+^ sympathetic neurons, Fig. 6C, right; Fig. 6D, gray bars). Compared to the 45% benchmark efficiency, the SAP only gave rise to 10% neurons, i.e. 10% of all cells in the dish were PRPH^+^ peripheral neurons (Fig. 6C, left and **6D**, light purple bar). However, of all those peripheral neurons, 95% were sympathetic in character, i.e. 95% of those PRPH^+^ cells expressed TH (Fig. 6C, left and **6D**, dark purple bar), comparable to the benchmark. Sympathetic neuron efficiency can be further improved to 75% TH^+^ by using aphidicolin, however this has not been applied to either of the differentiation potential experiments here^63^. This suggests that our SAP is not a bipotent SAP (as in **Figure S5**A), that can efficiently give rise to both ChrCs and sympathetic neurons. However, it might be a source of intramedullary sympathetic neurons, which are indeed a small percentage of all medulla cells.

We were further curious if SAP cells can generate SCPs and Schwann cells, which would indicate that they are not set in their developmental specification yet (Fig. 6A). Thus, we generated SAP cells and then subjected them to the SCP/Schwann cell differentiation protocol^33^ (Fig. 6E). We found that SAP cells can, but are not efficient at generating Schwann cells (~ 5% differentiation efficiency, Fig. 6F, left and **6G**, purple bar), especially when compared to Schwann cells generated from the Majd et al., protocol^33^ (Fig. 6F, right and **6G**, gray bar). This finding confirms *in vivo* reports^64^, showing that during development, the SCP split off from migrating neural crest cells before they express the SAP markers. Together, this data indicates that the SAPs are still somewhat plastic in their ability to generate some Schwann cells and some sympathetic neurons, but they do this at a very low efficiency, suggesting that their primary fate is to become ChrCs. This strengthens the notion that these cells might be the correct progenitor for the first wave of medullary ChrC differentiation.

### Investigation of the developmental potential of Schwann cell progenitor cells.

We next aimed at employing our human system to assess developmental ChrC findings, that have been reshaped in mouse and human systems in recent years. It is now known that early neural crest cells give rise to nerve-associated SCPs, which subsequently will travel along established preganglionic nerve tracks and populate between 75–80% of the adrenal medulla as SCP-derived ChrCs^15^ (Fig. 1A). It has also been described that SCPs have an extensive developmental potential, in that they are able to generate Schwann cells^64^, ChrCs^10,15^, parasympathetic neurons^58,65,66^, melanocytes^67,68^, and enteric neurons^69,70^, among other cell types^14^. It remains less clear if SCPs can give rise to intra- or extramedullary sympathetic neurons in humans^19^ and mice. Using the hPSC system, we showed that SCPs can differentiate into Schwann cells (Fig. 3C) and ChrCs (Fig. 3D–J). We also recently showed that hPSC-derived SCPs can generate parasympathetic neurons^58^.

We assessed the potential cross plasticity of our SCPs, investigating if they can give rise to sympathetic neurons, these might be extra- or intramedullary sympathetic neurons, which is indistinguishable in our system (Fig. 6H). To do so, we put d16 SCPs through our sympathetic neuron differentiation protocol^39^ (Fig. 6I). We show that the SCPs do not efficiently generate sympathetic neurons. Only ~ 7% of all cells generate peripheral neurons (PRPH^+^ over DAPI^+^ cells) (Fig. 6J, left **and 6K**, light blue bar), compared to the 45% generated from neural crest in the protocol^63^. Furthermore, of those neurons only ~ 7% have a sympathetic character, compared to the 90% generated from the neural crest in the protocol (Fig. 6K, dark blue bar). This suggests that of the few peripheral neurons that SCPs generate, most are of non-sympathetic character; they might be enteric or parasympathetic neurons^71^. Together, this data suggests that SCPs derived from hPSCs give rise to ChrCs and Schwann cells, but not sympathetic neurons, and thus are mimicking human embryonic development appropriately.

### Schwann cell progenitor-derived and sympathoadrenal progenitor-derived chromaffin cells secrete epinephrine and norepinephrine differently in response to stress.

Many mammals, for example rats, bovines, and humans, have distinct EPI-secreting and NE-secreting populations of chromaffin cells in their adrenal gland^16^. However, it is unclear if this job separation exists in humans or if most of the chromaffin cells have the capability to perform both functions. The EPI-secreting chromaffin cells are distinguished from NE-secreting chromaffin cells by the expression of PNMT, an enzyme responsible for converting NE into EPI. We assessed whether one of the progenitors (SAP or SCP) preferentially gives rise to EPI-secreting ChrCs by comparing the percentage of PNMT^+^ cells by fluorescent activated cell sorting (FACS) analysis. About 94% of ChrCs derived from either progenitor or a mixed progenitor population are PNMT^+^ and are therefore capable of secreting epinephrine (Fig. 7A). However, the FACS data does not clarify differences in expression (i.e. if one has higher PNMT expression and thus could make more EPI). Therefore, we looked at the mRNA expression of *PNMT* and *DBH* (the enzyme capable of making NE) and found that SAP-derived ChrCs express more PNMT, while SCP-derived ChrCs expressed more DBH (Fig. 7B). This indicates a difference in EPI/NE release between SCP-derived ChrCs and SAP-derived ChrCs. We sought to verify this by looking at the EPI and NE release in response to stimulation by the neurotransmitter ACh and the neuropeptide pituitary adenylate cyclase-activating polypeptide (PACAP), both of which are released by the preganglionic sympathetic neurons that innervates the adrenal medulla^54^ (Fig. 7C–E). In no-stimulation basal conditions, both progenitors gave rise to ChrCs with similar NE/EPI release. This suggests that the effects of the differential enzyme expression are irrelevant without stress. However, the differential enzyme expression becomes relevant and “limiting” under stress conditions, where enzyme activity is “maxed.” For the SAP-derived ChrCs, the higher PNMT expression made them more EPI responsive (Fig. 7C). Based on the DBH mRNA expression and the EPI/NE release data, DBH may limit the amount of EPI/NE secretion by the SAP-derived ChrCs (Fig. 7F). However, we hypothesize that the little NE produced by the small amount of DBH is turned into EPI due to the high amount of PNMT. For the SCP-derived ChrCs, they primarily release NE under stress conditions with a small amount of EPI being released (Fig. 7D). For the SCP-derived ChrCs, the limiting enzyme appears to be PNMT, reducing the amount of EPI secreted to amounts like the SAP, but since this limitation is further downstream, the SCP-derived ChrCs respond with very high release of NE. For the mixed progenitor ChrCs, the NE/EPI secretion profile is an intermediate between the SAP-derived and SCP-derived progenitors (Fig. 7E). Altogether, this suggests that, while a majority of either progenitor-derived ChrCs are capable of producing EPI, SAP-derived ChrCs may be capable of producing more EPI due to higher expression of PNMT, but may be limited upstream by lower DBH expression (Fig. 7F). On the flipside, the high DBH expression and low PNMT expression in SCP-derived ChrCs suggest that NE synthesis is not limited, but EPI synthesis would be (Fig. 7G). Taken together, SCP-derived ChrCs and SAP-derived ChrCs have a different catecholamine release profile under stress conditions due to differences in catecholamine synthesizing enzymes, suggesting that the reason for developing the medulla from two distinct progenitors may be to provide ChrCs with distinct functional responsibilities.

## Discussion

Over the past few years several papers have revisited the question of embryonic development of the adrenal gland medulla. Even though this remains a dynamically moving field, so far, using both mouse *in vivo* tracing studies and transcriptomic studies in human fetal adrenal gland tissue, it was concluded that the medulla has two embryonic progenitor origins. The early migrating neural crest cells give rise to the SAP, which then invade the adrenal cortex progenitor cells to establish the first wave (~ 20–25%) of ChrCs. Later in time, the neural crest-derived SCPs migrate along established preganglionic nerve tracks and populate the remaining ~ 75–80% of the adrenal gland medulla (Fig. 1A).

In this work, we aimed at employing the hPSC system to 1) generate human adrenomedullary organoids that can be employed for disease modeling and 2) to study human embryonic development and corroborate such *in vivo* findings. We defined *in vitro* differentiation protocols for the derivation of adrenomedullary organoids from those two embryonic sources, the earlier SAP cell progenitors (Fig. 1, 2, 4), and the later SCPs (Figs. 3 and 4). We show that adrenomedullary organoids can be derived from both sources and that they are both functionally active (Figs. 2 and 3).

Single cell transcriptomic analysis of our adrenomedullary organoids indicated the presence of various cell types, as well as cell subtypes within the embryonic adrenal gland (Figs. 4, S3 and S4). While several groups have performed scRNAseq. analyses on the human embryonic adrenal medulla, the majority of these analyses have been performed with the aim of understanding the developmental origin of cell types implicated in pediatric neuroblastoma^19,31,32,72^. These studies have provided numerous insights, leading to the identification of species-specific cell type markers, such as *CARTPT* as a marker of ChrCs in human versus mouse sympathoblasts^73–75^, or markers for adrenal medulla progenitors^72,76^. They supported a paradigm shift regarding the developmental origin of the majority of the adrenal medulla. However, these studies’ focus was not on the medullary subtypes^21,72^, thus the full scope of human ChrC subtypes and their functional roles have yet to be elucidated. Here, we identified a novel adrenomedullary subtype, that is exclusively derived from the SAP, the CLDN11+/AGTR1+/organoid ChrC1 cluster. These cells are transcriptionally similar to a novel ChrC-like cell described by Bedoya-Reina et al.^72^ as *NTRK2*+/*CLDN11*+. These cells expressed progenitor markers (*SOX6*, *BCL11A*) and the adrenergic marker *CHRNA7*, but did not express typical ChrC markers such as *TH*, *DBH*, or *CHGA*, and were not located within the medulla, but rather along its periphery and in the periphery of the adrenal gland under the capsule. We identified clusters co-expressing angiotensin II receptor type 1 (*AGTR1*) with *CLDN11* both in our organoids and in our re-analysis of an embryonic adrenal medulla scRNAseq. Furthermore, a cluster we identified as a progenitor for ChrC1 in our organoids expressed both *CLDN11* and *NTRK2*. Although *AGTR1* was not observed to be expressed by the ChrC-like cells in Bedoya-Reina et al., the observed localization of these cells to the adrenal periphery is intriguing. Given the importance of angiotensin II signaling in the outer aldosterone-secreting regions of both the developing and adult adrenal cortex, further studies are needed to explore the possibility that AGTR1-mediated signaling plays a role in development.

Additionally, we employed our adrenomedullary organoids to address the role that each ChrC type may play during the stress reaction. We suggest that the SAP-derived ChrCs may be responsible for severe stress, such as severe infection conditions and that they may react with more EPI, rather than NE (Fig. 7). This may be consistent with the SAP-derived novel ChrC1 cell population identified in our scRNA seq. data that is reminiscent of the ChrC-like cells in Bedoya-Reina et al.. On the other hand, our data suggests that the SCP-derived ChrCs may be responsible for the everyday demand reaction and that they do so via NE, rather than EPI (Fig. 7). Functional differences between ChrCs within the medulla have been described before. When ChrCs are separated based on their release of NE versus EPI, several lines of evidence have been presented: 1) There are indications that different stressors activate the different ChrCs. For example, public speaking stress activates more EPI release, while exercise stress leads to more NE release^77^. 2) The medulla is innervated by two branches of the preganglionic splanchnic nerve and evidence was presented that NE versus EPI releasing ChrCs react to different frequencies of preganglionic stimulation, i.e. higher stimulation (from both branches) leads to EPI release, while lower stimulation (from one branch) leads to NE release^78,79^. 3) The two types of ChrCs are innervated by different neuron fibers, based on electrophysiological and histological analysis^80,81^. In combination with our and previous results that the two distinct embryonic progenitors give rise to the medulla; it will be interesting to further investigate if the developmental origin of the cells correlate or dictate their specific innervation and ensuing functionality.

We injected the progenitors or the differentiated ChrCs into the chick embryo. The progenitors integrated and migrated to the appropriate area within the organism, and the ChrCs integrated into the chicken adrenal gland tissue (Fig. 5), indicating their capacity for integration *in vivo*.

We employed our adrenomedullary organoids to assess the developmental capacity and plasticity of the two distinct ChrC progenitors. We showed that the hPSC-derived SAP cell progenitor cells are relatively restricted in their fate, as they efficiently give rise to ChrCs, but not or only very inefficiently to sympathetic neurons and Schwann cells. This observation is consistent with the developmental paradigm shift from the bipotent SAP capable of giving rise to both sympathetic neurons to a more fate restricted ChrC progenitor^48^. We further assessed the plasticity of the hPSC-derived SCP. We found that our SCPs can give rise to Schwann cells (Fig. 3), ChrCs (Fig. 3, Fig. 4) and parasympathetic neurons^58^. However, SCPs are not able to efficiently generate sympathetic neurons (Fig. 6). Kameneva et al. found that intramedullary sympathetic neurons in the mouse are derived from SCPs^19^. Conversely, we found in the hPSC-based system that, while SAP can give rise to few sympathetic neurons, the SCPs do not give rise to sympathetic neurons despite giving rise to 95% of sympathoblasts (Fig. 4). This might be attributed to a mouse-human difference. In agreement with recent publications documenting the SCP origin of ChrCs, we observed by scRNAseq. that 98% of canonical ChrCs were derived from SCPs, while SAPs gave rise to a distinct cell type with to date unknown function. These findings support the current paradigm, that while SCP and SAP can both give rise to ChrCs, SCP can give rise to many more lineages, including Schwann cells and parasympathetic neurons. SAP however is restricted to ChrCs and a small proportion of sympathetic neurons. This makes the developmental potential of SAP and SCP distinct.

In sum, we provide a paradigm for the *in vitro* generation of adrenomedullary organoids from hPSCs, either via an early neural crest progenitor primed for ChrC differentiation, or via a SCP, a later neural crest-derived, plastic, multipotent progenitor cell type. These organoids express characteristic markers and are functional *in vivo* and *in vitro*, containing a variety of adrenomedullary cell types and cell subtypes, supported by our scRNA-seq analyses. We employ the neural crest progenitors to assess their plasticity and investigate recent developmental *in vivo* finding in the human system. In the future, such adrenomedullary organoids will be important for disease modeling, disease mechanism discovery, drug screening and testing, and assessment of developmental paradigms in the human system.

### Limitations of the study:

ScRNA-seq. analysis revealed populations of progenitor cells at d30. Conducting functional/identity assessments later in the differentiation, beyond d30, may present a more mature adrenomedullary profile. The cell preparation process for scRNA-seq may have also resulted in the loss of some cells that are more sensitive to the dissociation methods. Assessing multiple time points where the cells were exposed to stressors (in addition to the 15-minute time point) could provide interesting results. These experiments could provide a timepoint for when the media becomes saturated with catecholamines, or the cells become exhausted. This would be useful for future stressor studies with adrenomedullary organoids and studying stress-disorders using adrenomedullary organoids.

## STAR Methods

### Resource availability

#### Lead contact

Further information and requests for resources and reagents should be directed to and will be fulfilled by the lead contact, Dr. Nadja Zeltner (nadja.zeltner@uga.edu)

#### Materials availability

Research materials generated in this article will be distributed upon reasonable request and upon completion of Material Transfer Agreements (MTAs).

#### Data and code availability

All data generated or analyzed in this study are included in this article and its supplementary data file. Raw data points are available from the corresponding author on a reasonable request. scRNA-seq data will be accessible on GEO (submission in process). This paper does not report original code. Raw data will be deposited on Mendeley or a similar resource before publication.

### Experimental model and study participant details

#### Cell lines

Stem cell lines used here are listed in Zeltner et al.^82^ and Fattahi et al.^28^ hESC lines: H9-ESC, WA09 and H9 EF1::RFP. hiPSC lines: healthy C1, 35 y.o. female, generated from Coriell fibroblast AG02602^83^. Stem cell lines were cultured as previously described^39^. Cells were maintained in Essential 8 medium (Gibco A15170–01) on vitronectin coated (Thermo Fisher/Life Technologies A14700, 5 μg/ml) cell culture plates, and passaged using EDTA (Sigma ED2SS). This study employed human embryonic stem cell lines (WiCell WA09), the use of which was approved to the Zeltner lab by WiCell. The iPSC line employed in this work was reprogrammed from a human sample obtained through the public repository Coriell Research Institute.

### Method Details:

#### SAP differentiation:

For SAP, from days 0 to 4, hPSCs were differentiated as in Wu et al. 2020^39^. Briefly, cells were seeded in differentiation media containing Essential 6 Media (E6, Gibco A1516401) containing 10 μM ROCK inhibitor (Y-drug, Biogems 1293823), 10 μM SB431542 (SB, Bio-techne 1614), 1 ng/mL BMP4 (Bio-techne 314-BP/CF), 300 nM Chir99021 (Chir, Bio-techne 4423). On day 1, ROCK inhibitor was removed. On day 2, BMP4 was removed and Chir99021 concentration was changed to 750 nM. From days 4–9, cells were treated with 500 nM Retinoic Acid (RA, Sigma R2625), 80 ng/mL NRG1 (Peprotech 100–03), 80 ng/mL SDF1 (Peprotech 300–28A), 750 nM Chir, 10 μM SB, 10 ng/mL BMP4.

#### SAP to ChrC differentiation:

On day 10, cells were dissociated with Accutase (Fisher, NC9464543) into single cells and seeded into ultra-low attachment 24-well plates at 1×10^6^ cells/well to self-aggregate in spheroid media comprised of Neurobasal base media (Gibco 21103049) supplemented with 1% L-glutamine (Gibco 25030–081), 2% B-27 supplement (without Vitamin-A; Gibco 12587001), 1% N2 supplement (Gibco 17502048), 10 ng/mL FGF-2 (bFGF, Bio-techne 233-FB/CF), and 3 μM Chir99021^38^. On day 14, media was changed to ChrC medium, containing Dulbecco’s Modified Eagle Medium/F-12 (DMEM F12, Gibco 11330032), 5% Fetal Bovine Serum (Corning 35–010-CV), Glutamax (Fisher 35050061), 2% B-27 supplement, 100 uM ascorbic acid (AA, Sigma A4544), 10 ng/mL FGF2, 10 ng/mL BMP4, 80 ng/mL NRG1, 50 ng/mL NGF (Peprotech 450–01), 25 ng/mL IGF-2 (Bio-techne 292-G2), 0.01 mM dexamethasone (Sigma D2915), and 100 nM phorbol 12-myristate 13-acetate (PMA, Cayman 10008014).

#### 2D ChrC culture/Landing of spheroids:

Dissociation: Cells were dissociated using Accutase at 37°C for 20 minutes and then plated on PO (Sigma, P3655)/LM (R&D Systems, 3400-010-02)/FN (Corning, 47743-654) coated plates at a density of 100,000 cells per cm^2^.

Landing: Cells were landed by roughly pipetting them 2–3 times and then landing them on PO/LM/FN plates for 6 hours at 37°C before fixation.

#### SCP differentiation:

For SCP, from days 0 to 10, hPSCs were differentiated as in Wu et al. 2020^39^. From days 10 to 16, cells were treated with Neurobasal supplemented with 1% L-glutamine, 2% B-27 supplement, 1% N2 supplement, 10 ng/mL FGF-2, 3 uM Chir99021, and 10 ng/mL NRG1.

#### Nanofiber Assay for Schwann cells

On day 16, SCPs were dissociated using accutase and plated on PO/LM/FN coated plates with nanofiber inserts (Nanofiber Solutions, 242402) at 1×10^5^/cm^2^ density. Cells were fed with day 24–30 medium of the Schwann cell differentiation for 7–10 days.

#### SCP to ChrC differentiation:

On day 16, media was changed to ChrC media.

#### Generating mixed adrenomedullary organoids:

D14 SAP and D16 SCP spheroids were dissociated using accutase and mixed together at a ratio of 20:80 into ultra-low attachment 24-well plates at 1×10^6^ cells/well to self-aggregate overnight in ChrC media.

#### Testing developmental potential: SAP to make sympathetic neurons:

Detailed protocol can be found in our previous publications^39^. Day 10 SAP were dissociated using accutase and cultured on ultra-low attachment plates at 0.5×10^6^/well. From day 10–14, cells were cultured in medium that contains Neurobasal medium, B27, L-Glutamine, 3 μM Chir99021 and 10 ng/ml FGF2. On day 14, spheroids were dissociated using accutase and plated on PO/LM/FN coated plate s 1×10^5^/cm^2^ density. To induce sympathetic neuron differentiation, cells were fed with medium that contains Neurobasal medium, B27, L-Glutamine, 25 ng/ml GDNF, 25 ng/ml BDNF, 25 ng/ml NGF, 200 μM ascorbic acid, 0.2 mM dbcAMP and 0.125 μM retinoic acid (add freshly every feeding). Experiments were performed after day 30.

#### Testing developmental potential: SAP to make Schwann cells:

On day 10, SAP were dissociated using Accutase and cultured on ultra-low attachment plates at 1×10^6^/well. From days 10–16, cells were cultured in medium that contains Neurobasal medium, 2% B27, 1% N2 Supplement, 1% L-Glutamine, 3 μM Chir, 10 ng/ml FGF-2 and 10 ng/ml NRG1. On day 16, spheroids were dissociated and plated on PO/LM/FN coated plates in medium that contained Neurobasal medium, L-Glutamine, N2, B27, 20 ng/ml NRG1, 10 ng/ml FGF2 and 100 μM dbcAMP (Sigma D0627). Schwann cells were analyzed as early as day 30.

#### Testing developmental potential: SCP to make sympathetic neurons:

On day 10, cranial neural crest cells were dissociated using accutase and replated into ultra-low attachment plates at 1×10^6^/well and fed day 10–16 SCP media. On day 16, SCP spheroids were dissociated using accutase and plated on PO/LM/FN coated plates 1×10^5^/cm^2^ density, from which point on they were fed the sympathetic neuron media described previously. Experiments were performed after day 30.

#### RNA Extraction and RT-qPCR

RNA samples were collected with Trizol (Invitrogen 15596018). Isolated RNA was resuspended in nuclease-free H_2_O. cDNA synthesis was performed using 1 μg of RNA (iScript, Bio-Rad 1708841) on an Eppendorf X50s thermal cycler. RT-qPCR was performed using SYBR Green Supermix (Bio-Rad 1725272) with a Bio-Rad CFX96 Real-Time System and C1000 Touch thermal cycler and analyzed by CFX Maestro. For primers used in this study.

#### Immunofluorescence

Adherent cells were fixed in cold 4% paraformaldehyde (PFA, Thermo Scientific AAJ19943K2) for 20 minutes and washed 3 times in PBS (Corning 45000–436). Cells were then permeabilized in a solution of 0.3% triton-X (Triton X-100, Sigma X100), 3% donkey serum (Sigma S30), 1% BSA (Sigma A4503) in PBS for 40 minutes and washed three times. Primary antibodies were incubated in blocking buffer (same as permeabilization buffer without triton-X) overnight at 4°C and washed with PBS the following day. Secondary antibodies blocking buffer were incubated for 1 hour at room temperature. Cells were then treated with DAPI for 5 minutes and rinsed 3 times in PBS.

Embryos were fixed overnight at 4°C in 4% PFA in PBS, incubated in 30% sucrose (Fisher S25590) in PBS for 24 h, and embedded in Optimal Cutting Temperature (O.C.T., Tissue-Tek 4583). Cryostat sections (12 μm) were collected on slides and kept at −20°C. The slides were then permeabilized and blocked in a solution of 0.1% triton-X, 5% donkey serum, in PBS for 1 hour. Primary antibodies were incubated in permeabilization/blocking buffer overnight at 4°C and washed three times with PBS the following day. Secondary antibodies were incubated for 1 hour at room temperature.

Sections of fixed 3D organoids were incubated at 4°C overnight in primary antibodies were incubated. Slides were then washed 3 times and incubated with secondary antibody for 2 hours. Slides were then washed three times and mounted with mounting solution (Abcam 104139).

Fluorescence microscopy was performed on an Agilent Lionheart microscope. Image acquisition was performed using Gen5 software. Image quantification was performed in FIJI.

#### Calcium Imaging

For stimulation conditions, cells were incubated in either 50 nM Acetylcholine (ACh, Cayman 23829) or 50 μM KCl (Sigma P5405) for 15 minutes at 37°C.

Cells were washed with PBS and incubated in 2 μM Fluo-4-AM (Thermo Scientific F10489) in fresh medium at 37°C for 20 min. Cells were washed three times with PBS and incubated again at 37°C for 30 minutes with fresh medium. After the incubation, the cells were read using a BioTek fluorescence microplate reader at 440 nm excitation and 520 nm emission.

#### HPLC

Media sample collection: 30 wells of d30 ChrCs in 24-well plates were aggregated into 1 well and incubated in 300 μL of ChrC media. After 24 hours of incubation, the media was collected.

Media preparation: An aliquot of cell culture media was collected and rapidly spun down at 14,200 × g for 1 min, and the supernatant was transferred to a fresh 1.5-mL centrifuge tube. Equal volumes of antioxidant buffer-1 (AO-1) (5 mg/mL sodium metabisulfite (Fisher Scientific S244), 0.4 M 70% perchloric acid (Fisher Scientific A229), 10 mg/mL EDTA (Sigma E9884) in HPLC-grade water (Sigma 270733) were added to the sample aliquot. A synthetic catecholamine not endogenously present in biological samples (isoproterenol, 416 ng/mL, Sigma I5627) was added to the buffer as the internal standard to correct the analyte signal deviation. The samples were rapidly vortexed for 30 sec and left on ice for an additional 1 min for efficient deproteinization. The contents were then transferred to Spin-X Nylon centrifuge tube filters (Corning 8169) and spun down at 14,200 × g for 10 min. All sample processing was performed at 4°C or on ice. The filter was discarded, and the samples were stored at −80°C until HPLC sample injection.

HPLC-ECD Method Validation, Chromatographic conditions, and performance: For neurochemical analysis, a commercial mobile phase (MDTM, Thermo Scientific NC9777698) was utilized. The assay was established using a previously validated method for HPLC-EC detection to detect tyrosine and tryptophan-based metabolites (norepinephrine (NE, Sigma A9512), epinephrine (EPI, Sigma E4375), dopamine (DA, Sigma H8502), homovanillic acid (HVA, Sigma H1252), 5-hydroxyindoleacetic acid (5-HIAA, Sigma H8876) serotonin (5-HT, Sigma H9523), in cell lysates^84–88^. Samples were loaded in the auto-sampler (Thermo Scientific WPS-3000TSL) and injected at a volume of 20 μL. The analytes were separated isocratically with commercial mobile phase by reversed-phase C-18 column (ZORBAX Eclipse Plus 4.6×100 mm, Agilent 959996–902) at 0.6 mL/min for 21 min (pump, Thermo Scientific ISO-3100 SD). Detection of NE, Epi, DA, HVA, ISO, 5-HIAA and 5-HT was performed by ECD (ESA CoulArray 5600A) using the following potentials (Channel 1: 350mV (ESA Guard cell 5020), Channel 3: −150 mV and CH4: +220 mV (ESA Microdialysis cell 5014B). Method development and data integration and analysis were performed using Chromeleon (v. 7.1.3) and ESA CoulArray Software (v. 3.10). A 9-point standard curve with the following concentrations: 2.5, 5, 10, 15, 20, 25, 50, 100, 250 ng/mL was generated, and the peak-area for the respective analytes determined the concentration.

#### Single Cell RNA sequencing:

Day 30 ChrCs were dissociated using Accutase (20–30 minutes for SCP-derived ChrCs and 30–40 minutes for SAP-derived ChrCs). A detailed protocol of the single cell RNA-sequencing preparation has been described in our previous publication^58^. Single cell RNA sequencing library preparation was done with PIPseqTM T20 3’ Single Cell RNA Kit v4.0 from Fluentbio with 40,000 cells per reaction.

#### Single Cell RNA sequencing analysis

The data samples provided as output from the PIPseeker software (https://www.fluentbio.com/products/pipseeker-software-for-data-analysis/) and the publically available embryonic scRNAseq data were all analyzed using the R package Seurat v5.1 following standard practices recommended by Seurat documentation. Samples were first subject to quality control by filtering out cells containing fewer than 200 unique genes, greater than 8000 (organoids) or 9000 unique genes (embryonic) and cells with more than 10% (organoids) or 25% (embryonic) of reads mapping to mitochondrial genes^89^. Raw gene expression data were log-normalized using Seurat’s NormalizeData function. Highly variable features were then identified with the “vst” method, selecting the top 5,000 most variable genes. The dataset was scaled via ScaleData, including the regression of mitochondrial read percentage to mitigate effects of mitochondrial content on downstream dimensional reduction. Principle component analysis was then performed on the scaled data, followed by canonical correlation analysis integration. Integrated datasets were then subject to FindNeighbors, FindClusters, and RunUMAP. The principle component threshold for FindNeighbors and RunUMAP was set as smaller value between the following two criteria: (1) the point at which the cumulative variance of principle components is greater than 90% and variance explained by each principle component is less than 5%, and (2) the point at which standard deviation between consecutive principle components is less than 0.1% (https://github.com/hbctraining/scRNA-seq/blob/master/lessons/old-clustering_analysis_all_clustering_and_qc.md). Clusters were then annotated by running FindAllMarkers to identify differentially expressed genes using the Wilcoxon rank sum test, retaining only genes expressed in at least 1% of cells in that cluster and with a minimum of absolute value 0.25 log2FC. Differential gene expression lists for each cluster were compared to canonical cell type markers identified from the literature. Clusters that were determined to represent the same cell type based on differential gene expression analysis were grouped, and FindAllMarkers was run again to obtain final differential gene expression lists. For clusters that were subset and re-clustered, cells in the subset were re-analyzed as described above starting from NormalizeData. Trajectory analysis was performed using Monocle3.

Correlation analysis between two single-cell RNA sequencing (scRNA-seq) datasets was conducted using the Seurat R package. Raw RNA counts from both datasets were normalized to account for differences in sequencing depth and library size. Pseudo-bulk expression profiles were generated by calculating the average gene expression values for each cell type using the AverageExpression function in Seurat. To identify highly variable genes (HVGs), the top 2000 most variable genes were determined for each dataset using the FindVariableFeatures function. The intersection of HVGs between the two datasets was calculated to identify common genes. Both datasets were then scaled using only the shared highly variable genes to ensure comparability. The scaled expression matrices from both datasets were combined based on the shared genes, and a pairwise Pearson correlation analysis was performed on the combined scaled expression matrix to evaluate transcriptional relationships across cell types. This workflow is adapted from the methodology described in Butler et al^90^.

#### *In ovo* transplantation

Fertilized White Leghorn chicken eggs (Hy-line North America, Mansfield, GA) were incubated at 38.5–39°C and 50–80% humidity. Cells in suspension were injected using a pulled glass capillary into the lumen of the neural tube (trunk level) at HH stage 16 (E2.5) or to the mesonephros area at HH stage 23 (E4). Each embryo was injected with 5 μl of 16M cells/ml in cultures media (about 80k cells per embryo). 100 unit/ml penicillin (VWR 97063–708) in Hank's Balanced Salt Solution (Thermo Scientific 14185052) was added on top of the embryos and embryos were incubated for 36 hours prior to analysis.

#### H295R Stimulation

Adrenocarcinoma cells (H295R, NCI-H295R, ATCC: CRL-2128) were grown on 10 cm TC-treated dishes in DMEM/F12 media supplemented with ITS+ premix (Corning CB-40352) and 2.5% Nu-Serum (Corning CB-51000). H295R cells were passaged once per week using trypsin. ChrC-conditioned media was collected from ChrCs at 48 hours after the last media change. Stimulation was performed using 10 μM FSK (Toris 1099) or ChrC-conditioned media for 48 hours.

#### ELISA

ELISAs were performed for norepinephrine (NE, Eagle Biosciences EA633), epinephrine (EPI, Eagle Biosciences EA632), and cortisol (Arbor Assays K003-H1) following the manufacturer instructions. For NE and EPI ELISAs, media was stabilized using the ELISA kit antioxidant stabilizer buffer and ChrCs were stimulated with 50 nM Acetylcholine and 1 nM PACAP (Selleckchem S8415) for 15 minutes at 37°C. All media samples were frozen at −80°C immediately after collection. ELISAs were read and analyzed on a BioTek Synergy 2.

#### Quantification and Statistical Analysis

Data was collected from at least three biological repeats. Biological replicates are defined as independent experiments conducted from a new thaw or different passage of a cell line^91^. It has previously been shown that there is negligible variability between iPSC clones, so when we have used an iPSC cell line for an experiment here, we used a single clone^82^. Data was presented as a mean ± SEM, and a p value < 0.05 was considered to indicate a statistically significant difference. Two-tailed unpaired Student’s t-test (for comparison of two groups) or one-way ANOVA followed by Tukey’s test (for comparison of three or more groups) were conducted using GraphPad Prism software. Asterisks indicate statistically significant differences between groups. p values are indicated in the figures as asterisks or “ns”, as follows: *, P ≤ 0.05; **, P ≤ 0.01; and ***, P ≤ 0.001, ****, P ≤ 0.0001. Analysis and graphing was done with Prism 9.

## Supplementary Material

Supplementary Files

This is a list of supplementary files associated with this preprint. Click to download.

- B.Sup1adherentversusspheroidChrCV1201.png
- E.Sup2mixedChrCV1201.png
- G.Sup.Fig.3ChrCscrnaseqfinal2fonts01.png
- H.Supp.Fig.4RNAseqV1fonts01.png
- K.Sup5developmentcartoonV1201.png

## Figures and Tables

**Figure 1:**
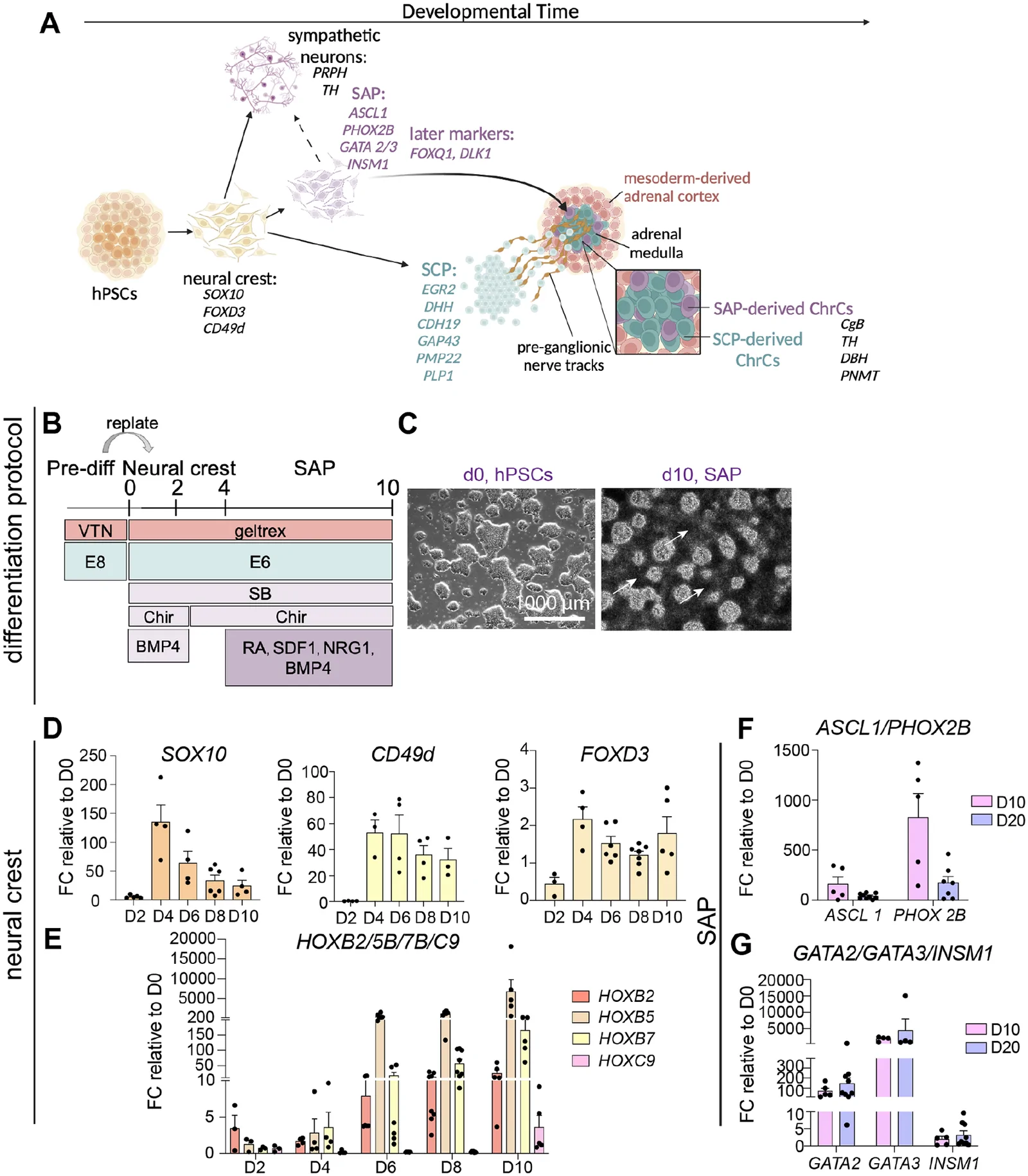
Human pluripotent stem cell-derived sympathoadrenal progenitor characterization **(A)** Schematic of adrenal medulla development with SAP-derived ChrCs and SCP-derived ChrCs, their gene markers at the neural crest, progenitor, and mature ChrC stage. **(B)** Differentiation protocol of SAP. **(C)** Phase contrast images of the differentiation: hPSC (day 0), SAP (day 10). White arrows point at neural crest ridges. **(D)** Progression of gene expression by RT-qPCR of *SOX10*, *CD49D*, and *FOXD3* during the neural crest/SAP stage of development between days 2–10. SOX10, n = 4–6; CD49d, n = 3–4; FOXD3, n = 3–8 biological replicates. **(E)** Progression of gene expression by RT-qPCR of *HOX2B/5B/7B/C9* during neural crest/SAP development between days 2–10. n = 3–8 biological replicates. **(F)** Progression of gene expression by RT-qPCR of *ASCL1* and PHOX2B of SAP/ChrCs between days 10–20. n = 5–10 biological replicates. **(G)** Progression of gene expression by RT-qPCR of *GATA2/3* and *INSM1* during SAP/ChrCs between days 10–20. n = 4–5 biological replicates. All data is presented as means ± SEM.

**Figure 2:**
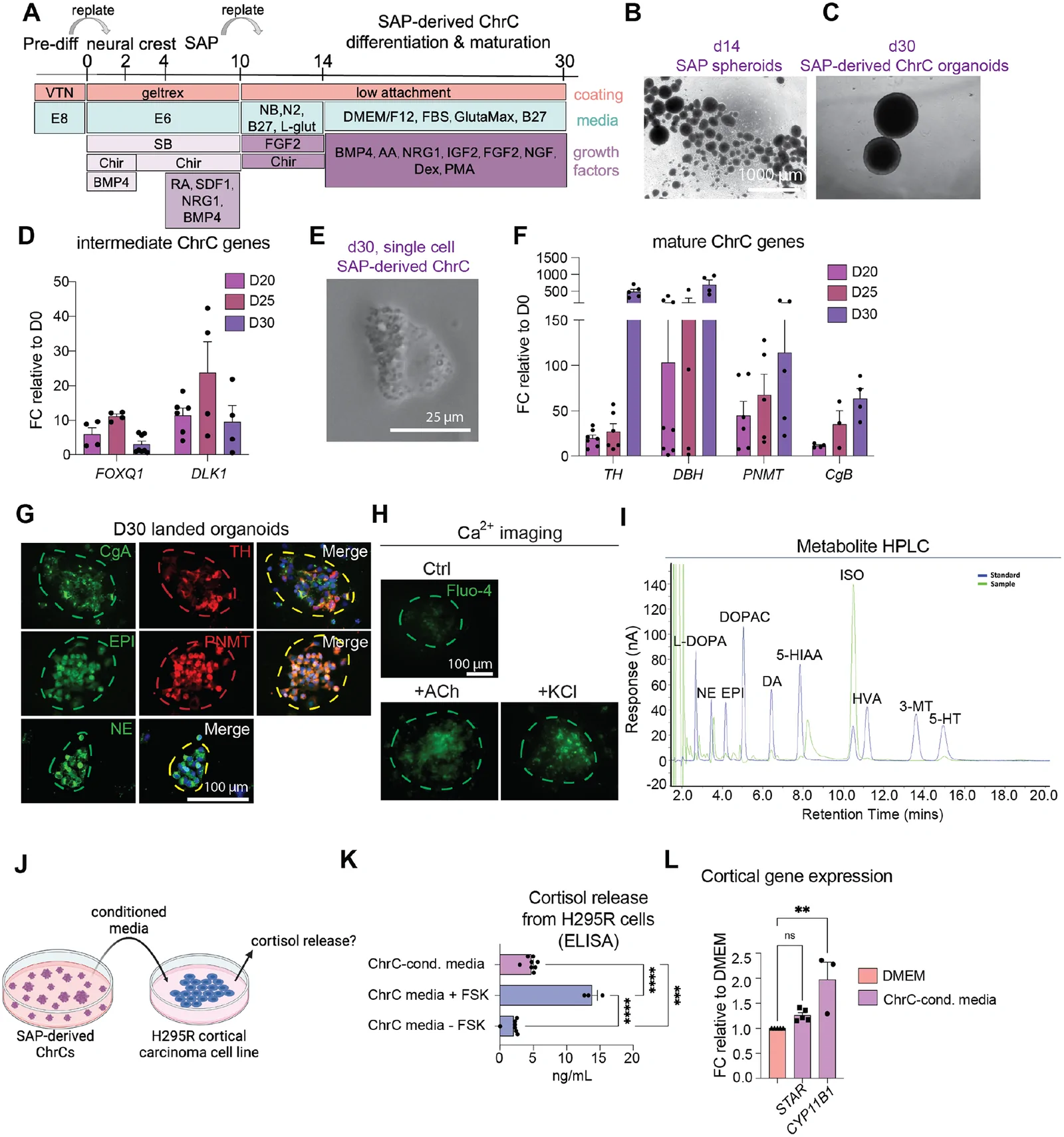
Sympathoadrenal progenitor-derived chromaffin cell characterization **(A)** Differentiation protocol of SAP-derived ChrCs. (**B**) SAP spheroids (day 14). (**C**) SAP-derived adrenomedullary organoids (day 30). (**D**) Progression of gene expression by RT-qPCR of *FOXQ1/DLK1* during intermediate SAP-derived ChrC development between days 20–30. n = 4–6 biological replicates. **(E)** Phase contrast image of a single cell adherent SAP-derived ChrC. Image was cropped. (**F)** Progression of gene expression by RT-qPCR of *TH/DBH/PNMT/CgB* during mature SAP-derived ChrC development between days 20–30. *PNMT* data is replotted from Figure S1. n = 3–8 biological replicates. **(G)** Expression of ChrC markers CgA, EPI, NE, PNMT, and TH in day 30 landed SAP-derived ChrC spheroids by immunofluorescence. **(H)** Calcium imaging of landed day 30 SAP-derived ChrC spheroids in response to stimulation by ACh and KCl. **(I)** Metabolite secretion by HPLC of L-DOPA, NE, EPI, DOPAC, DA, 5-HIAA, HVA, 3-MT, 5-HT of media incubated on SAP-derived ChrCs for 24 hours. **(J)** Schematic of experimental design to test if SAP-derived ChrC conditioned media could stimulate cortisol release from H295R cortical carcinoma cell line. **(K)** Measurement of cortisol release by ELISA in 3 groups: 1. SAP-derived ChrC conditioned media incubated on H295R cells for 48 hours; 2. ChrC media with FSK incubated on H295R cells for 48 hours (+ control); 3. ChrC media without FSK incubated on H295R cells for 48 hours; n = 3–9 biological repeats. One-way ANOVA, multiple comparison tests. ***p < 0.001, ****p < 0.0001. (**L)** H295R expression of steroidogenic genes *STAR* and *CYP11B1* by RT-qPCR incubated in either DMEM or SAP-derived ChrC conditioned media. n = 3–5 biological repeats. One-way ANOVA, multiple comparison tests. ns = not significant, p > 0.05, **p < 0.01. All data is presented as means ± SEM.

**Figure 3:**
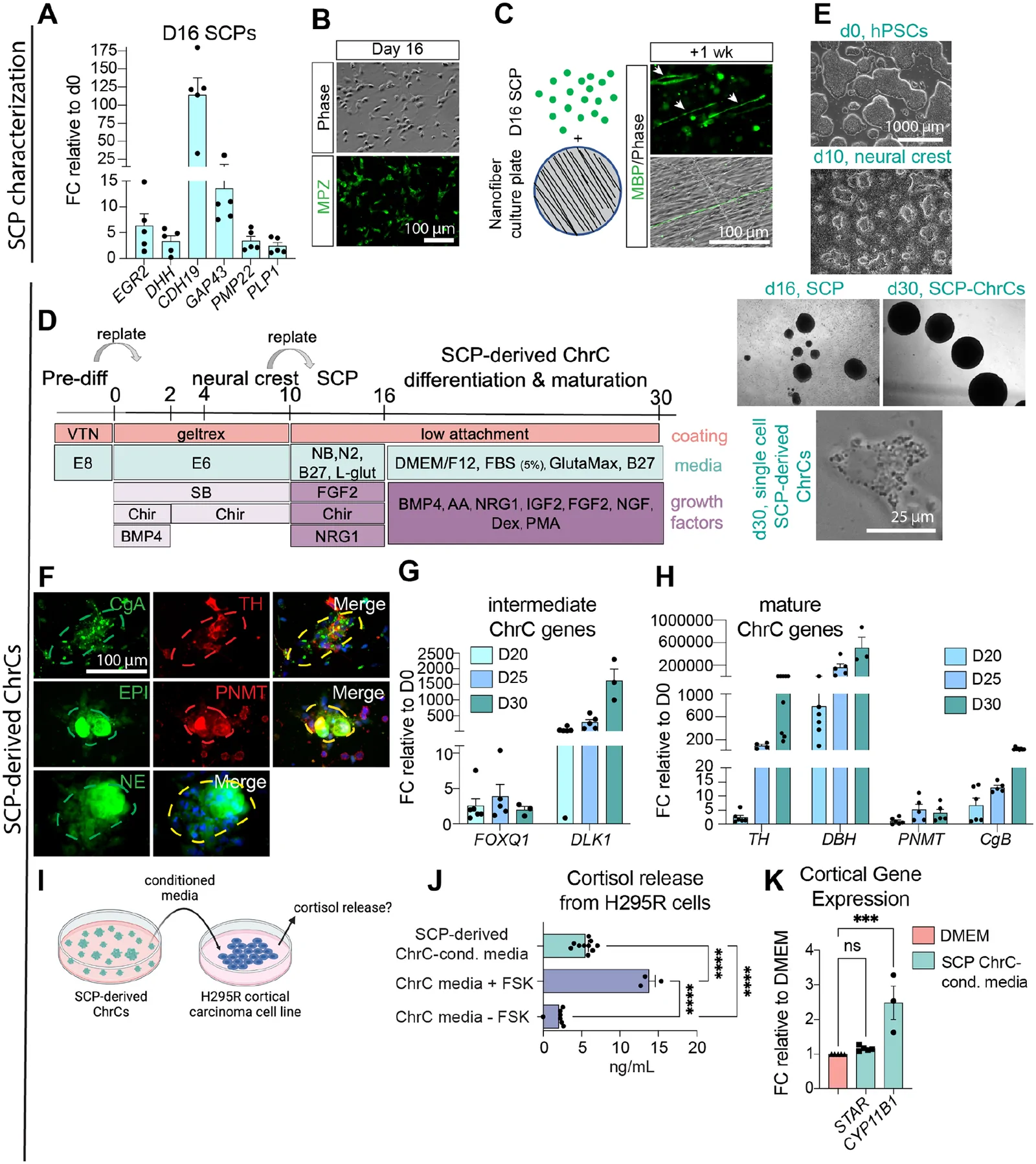
Schwann cell precursors give rise to chromaffin cells. **(A)** Expression of SCP gene markers on day 16 by RT-qPCR. n = 5 biological replicates. **(B)** Phase contrast (top) and expression of MPZ by immunofluorescence (bottom) in day 16 SCPs. **(C)** Expression of MBP in Schwann cells cultured on nanofibers for 1 week by immunofluorescence (top) and phase contrast (bottom) showing co-localization of MBP^+^ cells with nanofibers. **(D)** Schematic of SCP to ChrC differentiation protocol. **(E)** Phase contrast images of the SCP-derived ChrCs at different stages of development: hPSCs (day 0), neural crest (day 10), SCP spheroids (day 16), and SCP-derived ChrCs (day 30), phase contrast image of a single cell adherent SCP-derived ChrC (Image was cropped). **(F)** Expression of ChrC markers CgA, EPI, NE, PNMT, and TH in day 30 landed SCP-derived ChrC spheroids by immunofluorescence. **(G)** Progression of gene expression by RT-qPCR of *FOXQ1/DLK1* during intermediate SCP-derived ChrC development between days 20–30. n = 3–6 biological replicates. **(H)** Progression of gene expression by RT-qPCR of *TH/DBH/PNMT/CgB* during mature SCP-derived ChrC development between days 20–30. n = 3–9 biological replicates. **(I)** Schematic of experimental design to test if SCP-derived ChrC conditioned media could stimulate cortisol release from H295R cortical carcinoma cell line. **(J)** Measurement of cortisol release by ELISA in 3 groups: 1. SCP-derived ChrC conditioned media incubated on H295R cells for 48 hours; 2. ChrC media with FSK incubated on H295R cells for 48 hours (+ control); 3. ChrC media without FSK incubated on H295R cells for 48 hours. n = 3–10 biological repeats. One-way ANOVA, multiple comparison tests. ***p < 0.001, ****p < 0.0001. **(K)** H295R expression of steroidogenic genes *STAR* and *CYP11B1* by RT-qPCR incubated in either DMEM or SCP-derived ChrC conditioned media. n = 3–5 biological repeats. One-way ANOVA, multiple comparison tests. ns = not significant, p > 0.05, ***p < 0.001. All data is presented as means ± SEM.

**Figure 4:**
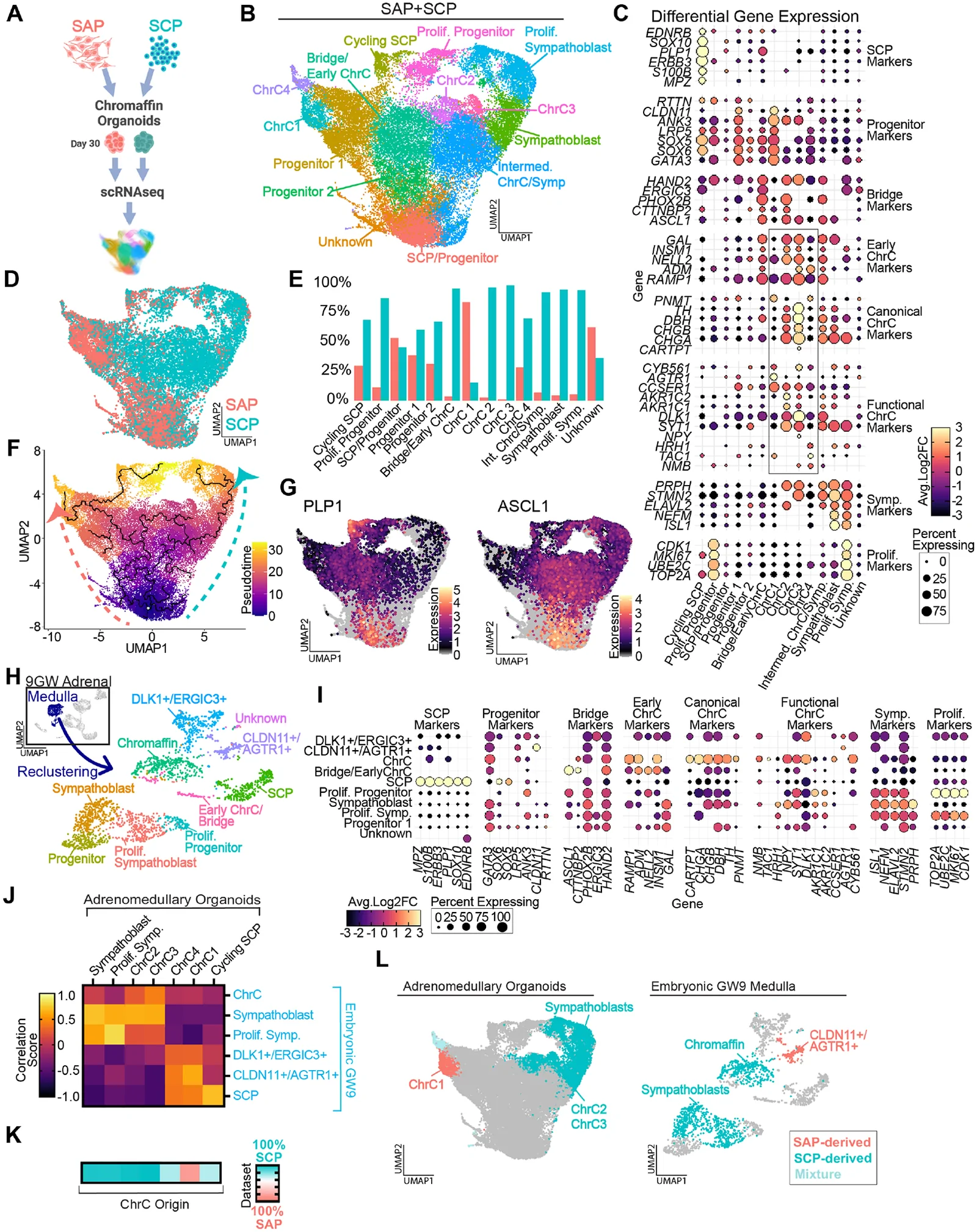
Single cell RNA sequencing analysis of adrenomedullary organoids from both progenitors **(A)** Overview diagram of scRNA-seq experiment. **(B)**Uniform manifold approximation and projection (UMAP) of visualization of cell types present in the integrated dataset, consisting of 9342 SAP cells and 27282 SCP cells (n=1). **(C)** Dot plot of differential gene expression (log2FC) for adrenomedullary cell marker genes used to identify cell types in B. **(D)** UMAP colored by the dataset each cell originates from. Pink = SAP, blue = SCP. **(E)** Bar plot showing the percent sample composition of each identified cell type. (**F**) Trajectory analysis showing the Monocle3-calculated trajectories from the *PLP1*+/*ASCL1*+ root identified in **G**. **(G)**UMAP projection of expression levels of medulla progenitor markers *PLP1* and *ASCL1*. Dashed lines indicate the trajectory of SAP (pink) and SCP (blue). (**H**) UMAP highlighting distinct cell types identified in a re-clustered 9GW embryonic medulla containing 2214 cells (subset of **Fig. S3D**). **(I)** Dotplot visualization of differential expression for markers of adrenal medulla cell types in **H**. **(J)** Heatmap of correlation analysis scores between embryonic cell types (rows, blue text) and organoid cell types (columns, black text). See also **Fig. S3G**. **(K)** Dataset progenitor origin (by percent) of organoid cell types in heatmap. (**L)** UMAPs of organoid and embryonic datasets with clusters colored by progenitor origins of adrenomedullary cell types.

**Figure 5:**
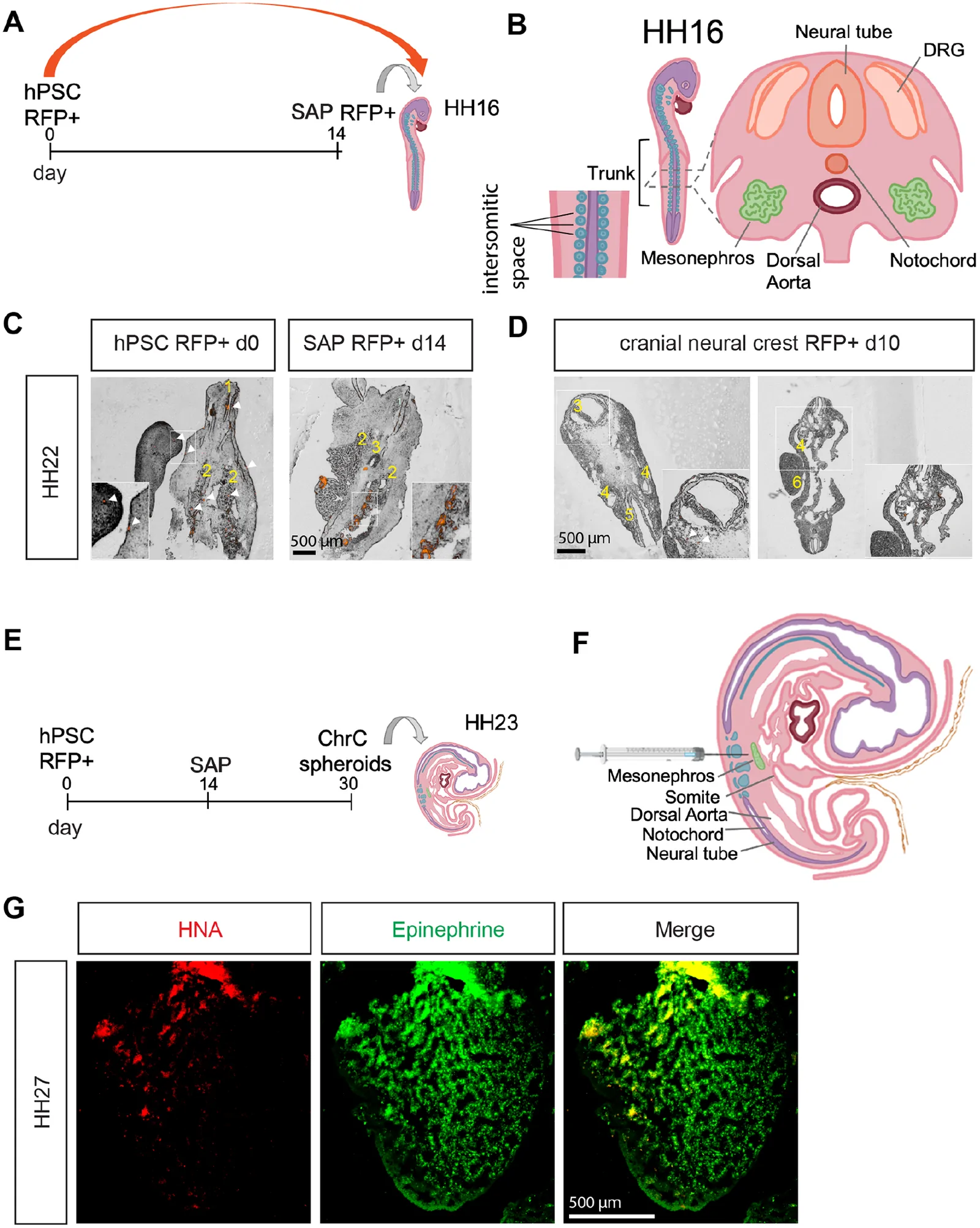
*In vivo* assessment of chromaffin cells in the chick embryo **(A)** Schematic of experiment where non- differentiated (day 0 RFP^+^ hPSCs) and differentiated cell types (day 14 SAP) were injected into the trunk region of chick embryos at developmental stage HH16. **(B)** Schematic of a section through the trunk region of a chicken embryo at HH16. **(C)** Sections of a chicken embryo trunk area at HH22 (36 hours after injection) injected with RFP^+^ hPSC (day 0) or RFP^+^ SAP (day 14). Arrowheads point to RFP^+^ cells integrated into the embryos. Labels are as follows: 1-Neural Tube, 2-Mesonephros, 3-wing/limb. **(D)** Sections of a chicken embryo’s head area at HH22, injected with RFP^+^ cranial neural crest (day 10). Labels are as follows: 4-skin, 5-myencephalon, 6-optic vesicle. **(E)** Schematic of experiment, where SAP-derived ChrCs (day 30) were injected into a HH23 chick as spheroids. **(F)** Cells were injected into the mesonephros region of the embryo. **(G)** Sections of chick embryo’s trunk area at HH27 co-immunostained with HNA (red) and EPI (green).

**Figure 6:**
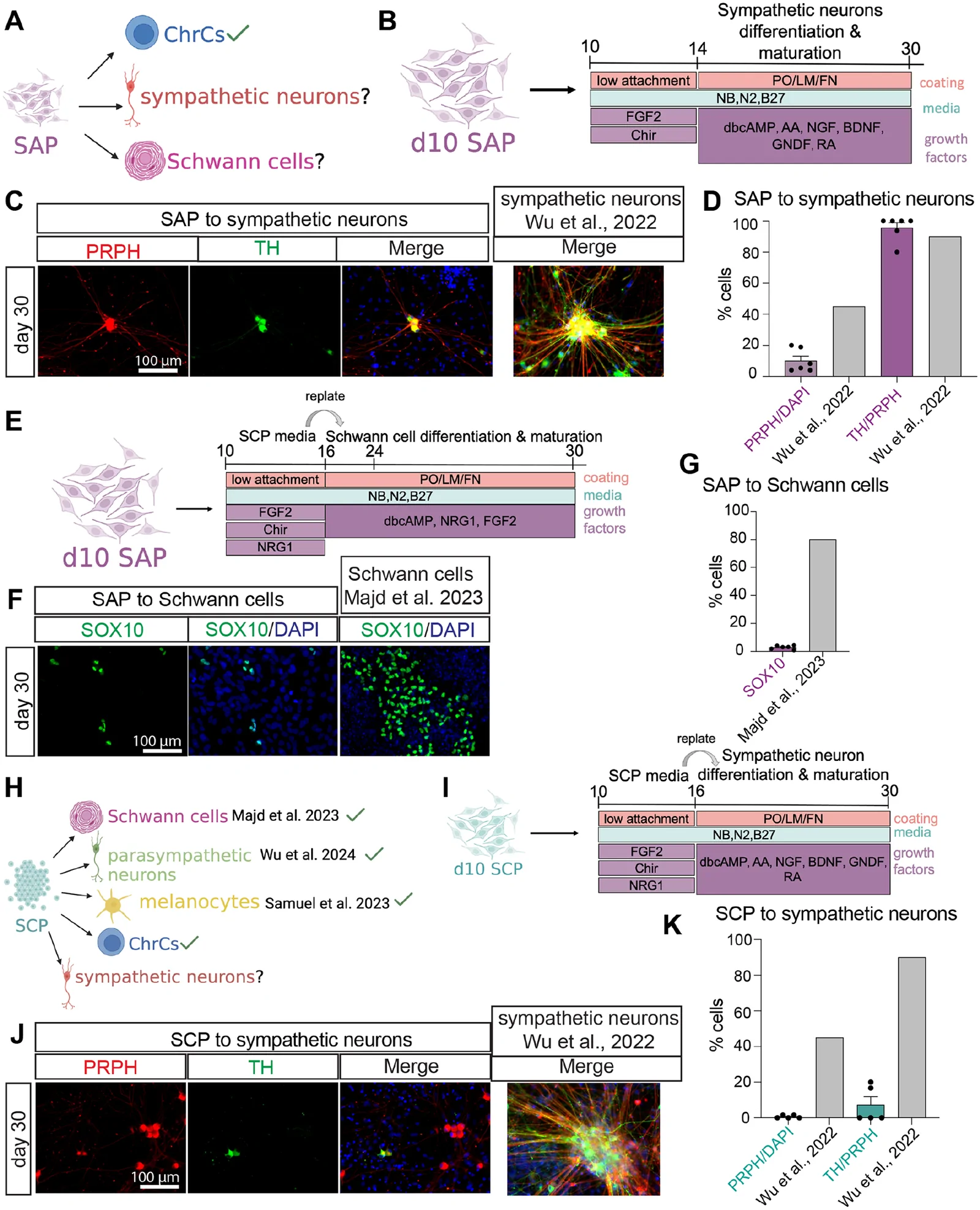
Assessing the developmental potential of the progenitors (**A**) Schematic of the developmental potential of SAP to ChrCs, sympathetic neurons, and Schwann cells. **(B)** Protocol to derive sympathetic neurons from day 10 SAP. **(C)** Immunofluorescent images of PRPH and TH in day 30 SAP-derived sympathetic neurons (left) and day 30 sympathetic neurons reproduced in our lab based on the Wu et al. 2022 protocol (right). **(D)** Quantification of immunofluorescent day 30 SAP-derived sympathetic neurons: percentage of neurons (PRPH/DAPI) compared to Wu et al. 2022 protocol & percentage of sympathetic neurons (TH/PRPH) compared to Wu et al. 2022 protocol. **(E)** Protocol to derive Schwann cells from SAPs. **(F)** Immunofluorescent images of SOX10 and DAPI in day 30 SAP-derived Schwann cells (left) and Majd et al. 2023 protocol derived Schwann cells for comparison (right). **(G)**Quantification of SOX10 in immunofluorescent images of SAP-derived day 30 Schwann cells (purple bar) compared to Schwann cells derived from Majd et al. 2023 protocol. **(H)** Schematic of developmental potential of SCP to Schwann cells, parasympathetic neurons, ChrCs, sympathetic neurons, and melanocytes. **(I)** Protocol to derive sympathetic neurons from SCP. **(J)** Immunofluorescent images of PRPH and TH in day 30 SCP-derived sympathetic neurons (left) and day 30 sympathetic neurons reproduced in our lab based on the Wu et al. 2022 protocol. **(K)** Quantification of immunofluorescent day 30 SCP-derived sympathetic neurons: percentage of neurons (PRPH/DAPI) compared to Wu et al. 2020 protocol & percentage of sympathetic neurons (TH/PRPH) compared to Wu et al. 2020 protocol. All data is presented as means ± SEM.

**Figure 7:**
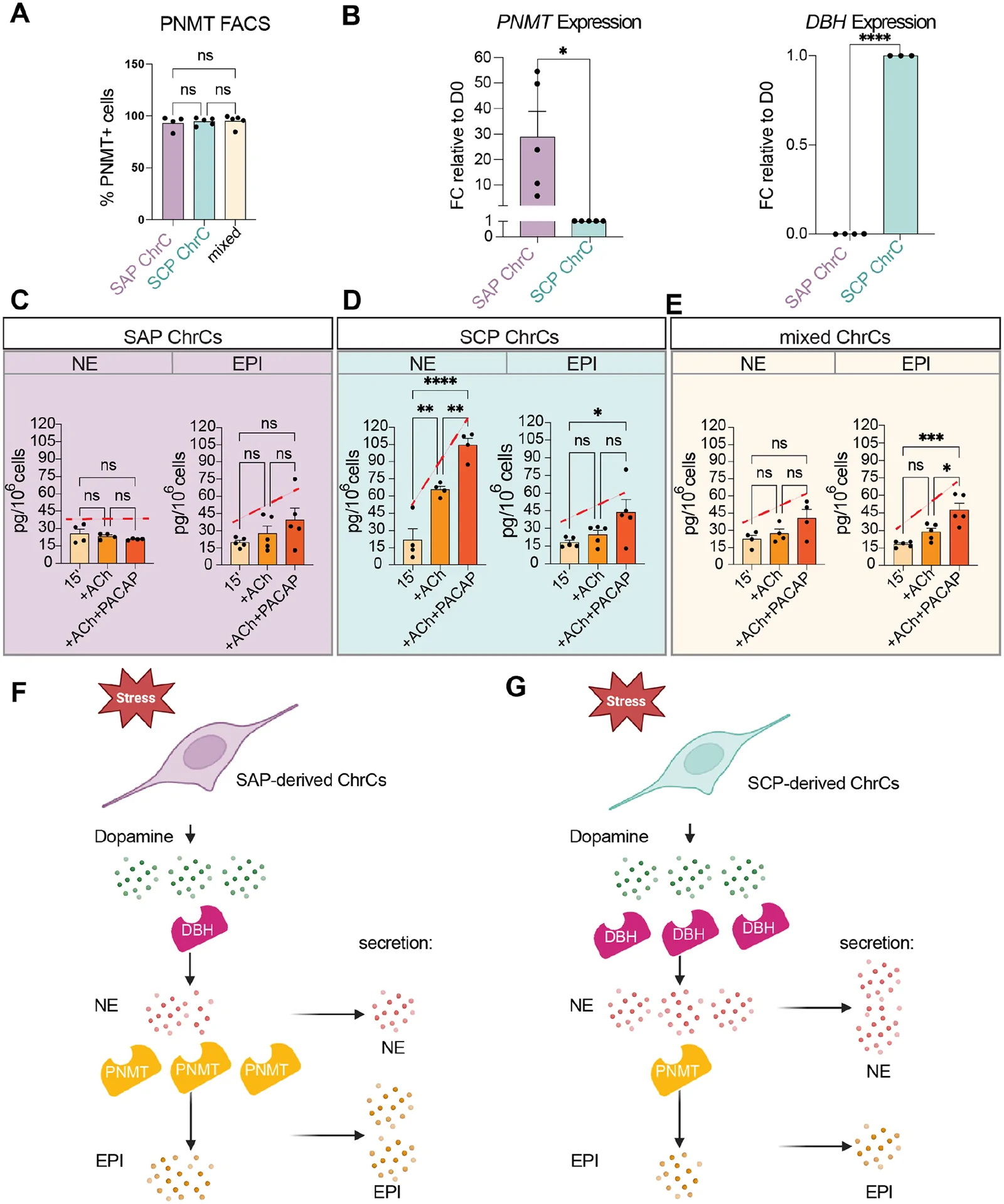
Chromaffin cells have differential stress induced secretion based on progenitor origin **(A)** Percentage of cells that are PNMT^+^ for SAP-derived ChrC organoids, SCP-derived ChrC organoids, and mixed ChrC organoids. n=5–6 biological replicates. One-way ANOVA, multiple comparison tests. ns = not significant. **(B)** Expression of *PNMT* via RT-qPCR for SAP-derived ChrCs and SCP-derived ChrCs. Data is replotted from Figure S1B and normalized by the mean of SCP-derived ChrCs. n= 5 biological repeats. Expression of *DBH* via RT-qPCR for SAP-derived ChrCs and SCP-derived ChrCs. Data is replotted from Figure 2F and normalized by the mean of SCP-derived ChrCs. n = 3–4 biological repeats. **(C-E)** NE and EPI secretion by ELISA after 15 minutes without stimulating factors, 15 minutes with ACh, or 15 minutes with ACh and PACAP stimulation. NE, n = 4 biological replicates. EPI, n = 5 biological replicates. Stimulation was done on SAP-derived ChrC organoids, SCP-derived ChrC organoids, and mixed ChrC organoids. One-way ANOVA, multiple comparison tests. ns = not significant p ≥ 0.05, *p < 0.05, **p < 0.01, ***p < 0.001, ****p < 0.0001. The dashed red lines represent the general trend. **(F, G)** Schematics of working model of stress-induced release of NE and EPI for SAP-derived ChrC. organoids and SCP-derived ChrC organoids. All data is presented as means ± SEM.
